# P.Re.Val.E.: outcome research program for the evaluation of health care quality in Lazio, Italy

**DOI:** 10.1186/1472-6963-12-25

**Published:** 2012-01-27

**Authors:** Danilo Fusco, Anna P Barone, Chiara Sorge, Mariangela D'Ovidio, Massimo Stafoggia, Adele Lallo, Marina Davoli, Carlo A Perucci

**Affiliations:** 1 Department of Epidemiology, Regional Health Service-Lazio Region, via Santa Costanza 53, Rome, 00198, Italy; 2 National Agency of Regional Health Services, via Puglie 23, Rome, 00187, Italy

## Abstract

**Background:**

P.Re.Val.E. is the most comprehensive comparative evaluation program of healthcare outcomes in Lazio, an Italian region, and the first Italian study to make health provider performance data available to the public.

The aim of this study is to describe the P.Re.Val.E. and the impact of releasing performance data to the public.

**Methods:**

P.Re.Val.E. included 54 outcome/process indicators encompassing many different clinical areas. Crude and adjusted rates were estimated for the 2006-2009 period. Multivariate regression models and direct standardization procedures were used to control for potential confounding due to individual characteristics. Variable life-adjusted display charts were developed, and 2008-2009 results were compared with those from 2006-2007.

**Results:**

Results of 54 outcome indicators were published online at http://www.epidemiologia.lazio.it/prevale10/index.php.

Public disclosure of the indicators' results caused mixed reactions but finally promoted discussion and refinement of some indicators.

Based on the P.Re.Val.E. experience, the Italian National Agency for Regional Health Services has launched a National Outcome Program aimed at systematically comparing outcomes in hospitals and local health units in Italy.

**Conclusions:**

P.Re.Val.E. highlighted aspects of patient care that merit further investigation and monitoring to improve healthcare services and equity.

## Background

Over the last two decades, there has been increasing interest in the development and implementation of outcome and process indicators. Such indicators encourage accountability and improvements in the quality of health care services; they also guide accreditation and health care planning interventions [1-6]. Public and private organizations, as well as research projects, have used different indicators for comparative evaluation of the performance of healthcare providers and professionals. Some have released their results to the public in the form of web-based reports that compare hospital quality [7-13].

In Italy, national and regional outcome research programs have been conducted, but there has been no systematic comparison of outcomes at the national level. The Mattoni-Outcome Project, funded by the Italian Ministry of Health [13], and the subsequent Progr.Es.Si. Project, have been the main national experiences in this field. The Mattoni-Outcome Project methodologies were the starting point for the Regional Outcome Evaluation Program, called P.Re.Val.E. [14,15], which was conducted in the Lazio region of Italy (5, 493, 308 residents [16]). P.Re.Val.E. is the most comprehensive comparative evaluation of regional healthcare outcomes and the only Italian study to publicly disclose performance data.

The objectives of P.Re.Val.E. were to define and evaluate outcome/process indicators in order to:

• compare the outcomes of health care provided by different hospitals or in different geographical areas for purposes such as accreditation, remuneration, and enhancing citizen empowerment;

• compare population subgroups (e.g. socioeconomic subgroups), especially for evaluating and promoting equitable service provision;

• identify the minimum volume of activity associated with the best treatment outcomes;

• promote internal and external auditing;

• identify critical areas in which to implement programs that improve health care quality;

• monitor trends in health care quality over time.

In order to highlight differences in the performance of local commissioning authorities, P.Re.Val.E evaluated outcomes according to patient residence. Importantly, direct standardization methods [17-20] rather than indirect standardization methods [5,7,10,20] were used for the comparative evaluation of outcomes. The aim of this report is to describe the P.Re.Val.E. program, including information sources, statistical methodologies, results for 2006-2009 (published online at http://www.epidemiologia.lazio.it/vislazio/vis_index.php, and the updated Italian version at http://www.epidemiologia.lazio.it/prevale10/index.php), and the impact of releasing performance data to the public.

## Methods

### Study design and data sources

We defined 54 performance indicators in different clinical areas that included treatment for cardiac, cerebrovascular, orthopedic, obstetric, respiratory, and digestive disorders [14]. Some were health outcome indicators, such as 30-day mortality after an episode of myocardial infarction or 30-day complications after cholecystectomy; others were process indicators for which an association with improved health outcomes has been already proven, such as intervention within 48 hours of hospital admission for hip fracture in the elderly. Finally, hospitalization rates for pathologies generally treated out of the hospital, such as diabetes, asthma, and influenza, were considered an indication of failure in primary care [21].

Most indicators were selected based on their previous use in international and national studies [7-19,21-24], while other indicators were developed in order to describe particular components of care or clinical pathways. A rationale and a detailed operative protocol were elaborated for each indicator according to a standard outline [14]. The indicators were defined using information collected from regional health information systems covering the whole Lazio population: the Hospital Information System (HIS), the Emergency Information System (EIS), the Mortality Information System (MIS), the Report Admission-Discharge for Rehabilitation (RADR), and the Hospital Deliveries Information System (HDIS). For detailed information about each, please see http://www.epidemiologia.lazio.it/vislazio_en/fonti.php.

The HIS database was the primary source for case selection and outcomes/comorbidities data. We also used information from the EIS database to better identify comorbidities and to estimate the time to death or time to surgery after arrival at the hospital (i.e. admission to the Emergency Department or to a hospital ward). Deaths during the study period were identified using the HIS, the EIS, and the MIS. The RADR database was used to identify admission to a rehabilitation center following hospital admission for stroke. Finally, we used the HDIS database for more accurate identification of new births, primary cesarean deliveries, and risk factors for cesarean section which are not included in the HIS.

HIS records were linked with EIS, MIS, RADR, and HDIS records using deterministic record-linkage. To ensure maximum coverage of the population while avoiding double counting, the linkage method uses a unique patient identifier deriving from information on persons' names, date and place of birth and gender, according to Italian privacy legislation.

### Study population

P.Re.Val.E. analyzed hospital discharges in the Lazio region in the 2006-2009 period [14]. Most data were expressed as ratios in which the numerator represents the number of treatments/interventions provided or the number of patients with a given outcome (i.e., short-term mortality, hospitalization for specific conditions, etc.) and the denominator represents the group of patients at risk. In other cases, the indicators were defined as survival/waiting time (e.g., the wait for surgery after hip fracture).

The analyses were performed, two years over two years, by area of residence regardless of the hospital at which the patient was treated and by hospital.

### Definition and attribution of outcome

The outcome measures were as follows: 30-day mortality, short-term re-hospitalization, hospitalization for specific conditions, surgical procedures, short-term complications of specific interventions, and waiting times. The outcomes under study were attributed to the first emergency department at which the patient was treated (first access) or to the first admission hospital and to the area of residence.

### Coexisting medical conditions

Chronic comorbidities and/or severity characteristics that were potentially associated with the outcomes under study were chosen using information in the literature [7-9,17,18,21-23,25-30] and in the Mattoni-Outcome project [13]. The potential risk factors were identified on the basis of ICD-9-CM codes registered either during hospitalization for the condition under study (index hospitalization) or in previous hospital or emergency department admissions during the previous two years. Acute events occurring during the index hospitalization, which could be complications of care/treatments (i.e. on the causal pathway between exposure and outcome), were not included.

### Statistical analysis

Statistical analyses were performed by first admission or access hospital as well as by area of residence. Since patient characteristics, such as age, sex, severity of disease, and/or chronic comorbidities, could be heterogeneously distributed across the hospitals/areas of residence, risk-adjustment methods were applied [31]. The risk adjustment procedure involved construction of a severity measure ("a priori" risk) that was specific for the study population and the use of this measure to obtain "adjusted" outcome measures for comparison between hospitals/areas. The severity measure was calculated by analyzing the multivariate relationship between possible outcome predictors and the outcome considered by application of multivariate regression models (predictive models) including: 1) *a priori *risk factors (age, sex, and/or severity of the condition being investigated); and 2) factors selected by a bootstrap stepwise procedure. Stepwise analysis was performed with 500 replicated samples of the original data and significance thresholds of 0.10 and 0.05 for entry and removal, respectively.

Only risk factors (pre-existing chronic conditions, etc.) selected in at least 50% of the runs were included in the final models

We used logistic regression models for dichotomous outcome variables and survival models for outcomes expressed in terms of survival times. In order to estimate adjusted group-specific (hospital/area of residence) log odds of outcome, logistic regression models with no intercept and centred covariates were applied for each outcome. Adjusted risks were obtained for each group by back-transforming parameter estimates with the following formulas:

Adjrisk = [exp(estimate)/(1 + exp(estimate))]*k

where k is a correction coefficient introduced to take into account the nonlinear nature of the logistic model. K is calculated as follows:

$$k=\frac{actual\text{\_}number\text{\_}of\text{\_}events}{\overset{m}{\underset{j=1}{\sum }}p_{j}*n_{j}}$$

where p_j _are the adjusted risks, n_j _is the group size, and m is the number of groups.

This approach allowed comparison of the outcome for a given facility or area of residence with that of the whole study population and with each of the other facilities/areas [18,32,33]. In order to assess for cluster effects, we used the cluster sandwich ("robust") variance-covariance estimators, relaxing the usual requirement that the observations are independent. According to this methodology, the observations are independent across groups but not necessarily within groups.

The adjusted RR estimated for each hospital/area of residence, the adjusted risk or median waiting time, and the corresponding p-value were reported on-line in tabular and graphical forms.

For each indicator, trend analyses and comparisons of the 2008-2009 data versus the 2006-2007 data were developed by hospital and area of residence.

Variable Life-Adjusted Display (VLAD) charts [34,35] were constructed to identify the principal increases/decreases and trend reversals in the cumulative sums of the differences between the number of events observed (deaths, rehospitalizations, reoperations, complications, etc.) and the number expected based on the predictive model. Two tests of statistical significance were performed, the first to determine whether during the study period, the number of events observed in one month (from the second day of the previous month to the first day of the following month) was significantly different from the number of expected events and the second to determine whether the number of observed events between 2 trend-reversal points was significantly different from the number of expected events. Both significance tests were performed using the ratio of observed to expected events assuming a Poisson distribution.

Finally, since higher activity volumes of the hospitals are often associated with better outcomes, we also investigated those relationships (displayed as scatter plots).

The level of statistical significance was set at 5% (p < 0.05), and all analyses were performed using SAS Version 8.2 [36].

### Preliminary disclosure of performance data to clinicians and providers

Before public release of data, we shared the P.Re.Val.E methods and results with different groups of clinicians and providers, to promote discussion and encourage contributions and critical assessments.

## Results

We calculated 54 indicators, 9 of which were prevention quality indicators, that covered almost 40% of all hospital admissions in the Lazio Region during the 2008-2009 period. The results obtained for all indicators, available online at http://www.epidemiologia.lazio.it/prevale10/index.php, showed a great heterogeneity of the healthcare quality in the Lazio region.

Public disclosure of the indicators' results caused mixed reactions but finally promoted discussion and refinement of some indicators, e.g., for 30-day mortality after aortic aneurysm. These meetings led to new estimates for some indicators and stimulated audit activities among clinicians and healthcare organizations.

In 2008, Agency for Public Health of Lazio designed a clinical pathway for elderly patients with hip fracture. The clinical pathway was tested in five selected hospitals before the implementation in all Lazio hospitals.

In 2009, a regional health service regulation went into effect that required Lazio hospitals to adopt the clinical pathway for elderly patients with hip fracture and that introduced a compensation system for hospitals based on quality of healthcare (as in a pay-for-performance model). The DRG reimbursement rate for Lazio providers was linked to hospital performance. In fact, from 2009 on, the full DRG rate has only been paid for patients who underwent surgical treatment within 48 hours after admission, while rates for interventions performed after 48 hours were reduced proportionally based on the time to surgery.

Based on the P.Re.Val.E. experience, the Italian National Agency for Regional Health Services has launched a National Outcome Program aimed at systematically comparing outcomes in hospitals and local health units in Italy.

As an example, results pertaining the 30-day mortality rate after hospital admission for acute myocardial infarction (AMI) in Lazio region were shown [for the operative protocol see: additional File 1, Appendix: operative protocol].

**Figure 1:**
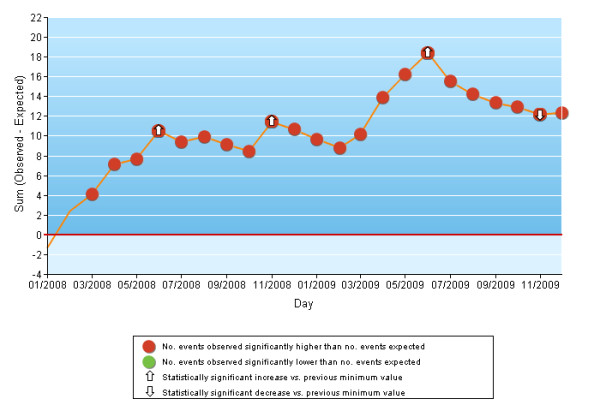
**VLAD chart**. Acute myocardial infarction: mortality within 30 days of hospital admission. Hospital Presidio Ospedaliero Nord, Latina.

**Figure 2:**
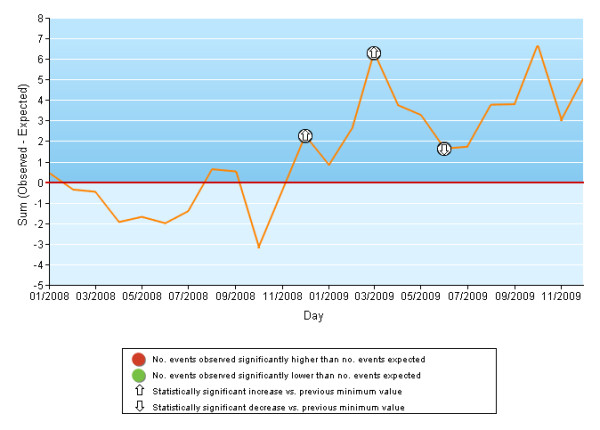
**VLAD chart**. Acute myocardial infarction: mortality within 30 days of hospital admission. Hospital S. Filippo Neri, Rome.

There were 16, 682 AMI episodes in 2008-2009 with a mean mortality rate of 11.1% (men: 8.9%; women: 15.2%). The probability of death was 2 times higher when chronic diseases (liver, pancreas, intestine) in the index admission, cancer and other cardiac operations were present (Table 1). The adjusted mortality rates ranged from 6.7% to 19.2% in different hospitals, and from 8.8% to 13.7% for different areas of residence (Tables 2 and 3). In the previous 2-year period there were 17436 AMI episodes, the mean mortality rate was 12.2%, with high variability among the different facilities and areas. In general, the adjusted mortality rates showed a small decrease over time for most facilities and areas (data not shown). Although the VLAD charts were developed for each facility and area, the results from two large hospitals that showed very different time trends for 30-day mortality after AMI admission were reported only. In 2008-2009, Presidio Ospedaliero Nord, Latina, showed a greater number of observed deaths after AMI admission than expected, with a little decrease in November 2009 (Figure 1), whereas San Filippo Neri, Rome, generally had fewer observed events than expected ones (Figure 2).

**Table 1:** Acute myocardial infarction: mortality within 30 days of hospital admission, predictive model

Risk Factors	N	Crude OR	Adjusted OR	p
Age (years)	-	1.08	1.08	0.000
Gender (Females vs Males)	5 767	1.84	1.05	0.419
Cancer	810	2.52	2.05	0.000
Hypertention	3 072	1.52	0.85	0.024
Previous myocardial infarction	2 481	0.96	0.73	0.000
Cardiomyopathy (index admission)	259	0.82	0.63	0.040
Cardiomyopathy	218	1.88	1.14	0.505
Heart failure	1 117	2.77	1.62	0.000
Other heart conditions (index admission)	367	1.06	0.60	0.006
Other heart conditions	228	1.83	1.08	0.688
Cerebrovascular disease	1 122	2.43	1.47	0.000
Vascular disease	679	2.23	1.57	0.000
Chronic renal disease	876	2.82	1.66	0.000
Other chronic disease (liver, pancreas, intestine) (index admission)	105	2.52	2.33	0.001
Other chronic disease (liver, pancreas, intestine)	209	1.98	1.35	0.135
Previous coronary angioplasty	1 417	0.57	0.65	0.000
Other cardiac interventions	127	1.69	2.06	0.006

**Table 2:** Acute myocardial infarction: mortality within 30 days of hospital admission, by health care facility

Hospital	Location	N	Crude rate × 100	Adjusted rate × 100	Adjusted RR	p
OSP. S. CAMILLO DE LELLIS	RIETI	509	12.57	12.23	1.10	0.461
OSP. S. SPIRITO	ROME	574	10.28	8.43	0.76	0.049
OSP. ANZIO-NETTUNO	ANZIO	351	9.69	9.11	0.82	0.269
OSP. ALBANO-GENZANO	ALBANO	361	13.02	12.75	1.15	0.366
OSP.S. PAOLO	CIVITAVECCHIA	298	14.77	13.86	1.25	0.162
OSP. PARODI DELFINO	COLLEFERRO	320	8.75	8.23	0.74	0.129
OSP. S. SEBASTIANO	FRASCATI	250	12.80	11.99	1.08	0.675
OSP. S. GIOVANNI EVANG	TIVOLI	200	19.50	19.17	1.73	0.001
OSP. GRASSI	ROME	648	9.72	9.17	0.83	0.152
OSP. S. EUGENIO	ROME	485	13.81	12.25	1.10	0.454
OSP. S.PIETRO F.B.F.	ROME	232	12.93	14.02	1.26	0.216
OSP. VANNINI	ROME	706	10.62	10.89	0.98	0.874
CC S. ANNA	POMEZIA	179	12.29	17.45	1.57	0.034
CC NUOVA ITOR	ROME	258	14.73	10.60	0.96	0.792
CC CITTA' ROMA	ROME	203	8.37	6.72	0.61	0.046
CC AURELIA HOSPITAL	ROME	396	10.35	10.59	0.95	0.775
PRES. OSP. NORD	LATINA	690	11.30	13.49	1.22	0.096
PRES. OSP. SUD	FORMIA	500	11.80	13.16	1.19	0.204
CC CITTA' DI APRILIA	APRILIA	245	13.47	13.48	1.22	0.286
OSP. UMBERTO I	FROSINONE	556	9.17	10.07	0.91	0.502
OSP. CIVILE	ANAGNI	152	9.87	10.98	0.99	0.968
OSP. S.S.TRINITA'	SORA	220	10.91	11.44	1.03	0.885
OSP. G. DE BOSIS	CASSINO	222	9.46	8.85	0.80	0.323
OSP. S. PERTINI	ROME	763	11.40	11.99	1.08	0.491
OSP. BELCOLLE	VITERBO	602	10.96	12.40	1.12	0.386
A.O. S. CAMILLO-FORLANINI	ROME	1021	12.83	13.00	1.17	0.088
A.O. S. GIOVANNI	ROME	679	7.81	7.52	0.68	0.007
A.O. S.FILIPPO NERI	ROME	770	10.91	11.58	1.04	0.708
POLICLINICO A. GEMELLI	ROME	695	8.06	7.73	0.70	0.011
POLICLINICO UMBERTO I	ROME	557	10.23	11.88	1.07	0.619
A.O. S. ANDREA	ROME	615	4.88	6.16	0.55	0.001
A.O. POL. TOR VERGATA	ROME	505	5.94	9.67	0.87	0.451

RR: Relative Risk

**Table 3:** Acute myocardial infarction: mortality within 30 days of hospital admission, by area of residence

Area	N	Crude rate × 100	Adjusted rate × 100	Adjusted RR	p
Rome District I	385	10.39	9.31	0.84	0.297
Rome District II	312	10.90	8.80	0.79	0.202
Rome District III	129	15.50	12.39	1.12	0.641
Rome District IV	567	9.70	9.04	0.82	0.152
Rome District V	709	10.86	11.13	1.00	0.982
Rome District VI	515	11.65	11.06	1.00	0.981
Rome District VII	298	9.40	9.86	0.89	0.546
Rome District VIII	565	9.03	10.96	0.99	0.932
Rome District IX	351	12.25	10.06	0.91	0.548
Rome District X	454	9.69	10.44	0.94	0.696
Rome District XI	415	14.70	12.49	1.13	0.387
Rome District XII	427	11.94	11.26	1.01	0.922
Rome District XIII	700	11.43	10.92	0.98	0.891
Fiumicino	221	9.50	9.90	0.89	0.620
Rome District XV	445	11.46	10.31	0.93	0.623
Rome District XVI	424	12.97	11.52	1.04	0.794
Rome District XVII	268	13.43	10.19	0.92	0.636
Rome District XVIII	393	12.72	11.46	1.03	0.827
Rome District XIX	594	11.62	10.05	0.91	0.444
Rome District XX	414	11.84	12.84	1.16	0.329
ASL RM/F	894	10.07	10.04	0.90	0.368
ASL RM/G	1278	10.95	11.78	1.06	0.500
ASL RM/H	1693	10.16	10.92	0.98	0.846
Viterbo	182	9.34	8.77	0.79	0.347
Viterbo Province	741	12.42	13.08	1.18	0.132
Rieti	181	9.39	9.37	0.84	0.504
Rieti Province	329	13.37	13.16	1.19	0.281
Latina	307	11.73	12.45	1.12	0.511
Latina Province	1223	11.37	12.73	1.15	0.124
Frosinone	180	11.67	13.71	1.24	0.345
Frosinone Province	1088	9.93	10.62	0.96	0.662

RR: Relative Risk

## Discussion and Conclusions

Comparative evaluation of hospital performance is a useful tool for improving health care quality [1,4]. The U.S. experience has shown that public disclosure of comparative evaluation results should be managed as one component of an integrated quality improvement strategy, and that the public release of performance data is most effective at the level of the provider organization [3,4,37]. Regular feedback seems to increase the accountability of providers, which are sensitive to public image and potential legal risks; it can also spur quality improvement activities in health care organizations, especially when underperforming areas are identified [38]. However, providers that are identified as poor performers are more likely to question the validity of the data, particularly when the results are first released [39].

In Italy, initiatives aimed at assessing the outcomes of hospital care have been undertaken at the national and regional levels only in the last decade [13,17,40,41]. Based on these experiences, we developed the Regional Outcome Evaluation Program, called P.Re.Val.E. [14]. The high numbers of patients investigated, the accuracy in the selection of the cohorts and the study outcomes, the consolidated statistical strategy, and the replication of similar findings for different clinical conditions are important elements of internal and external validity. For 2006-2009, results were obtained using direct risk adjustment for comparative evaluation of outcomes for hospitals and areas of residence. Hospital league tables obtained by indirect standardization procedures should not be used for hospital-to-hospital comparisons [20]. This technique can lead to biased conclusions unless the distribution of risk factors or their effects do not vary between the hospitals being compared.

After receiving comparative reports based on standardized performance measures, hospitals that began as low-level performers tended to improve faster than those that started at higher levels of performance [42].

Since studies have shown little correlation between measured quality of care and Standardized Mortality Ratios (SMRs) [43,44], we used different outcome and/or process measures for each studied condition to better identify the hospitals/geographic areas that needed health care quality improvement. This study used data from several health care information systems, and most of the indicators were based on the concept of first hospital access, corresponding to patient admission to an acute inpatient facility or to emergency department access. The indicators for which time to death or to surgery was calculated from first hospital access provide a measure of the appropriateness and efficacy of the health care process that begins when a patient arrives to a given facility. Use of the MIS and EIS databases, in addition to the HIS database, allowed more accurate identification of 30-day mortality.

To monitor the time trends of the different outcomes, we used VLAD charts, a type of quality control chart that is a good tool for measuring the variability of an event adjusting for patient risk [34,35,45,46]. VLAD charts provide an easy-to-understand and up-to-date view that allows early detection of runs of good or bad outcomes and thus can prompt timely intervention for critical situations. The VLAD charts also highlight small variations over time for observed events compared to expected events; this information is often obscured by the corresponding synthetic indicator.

The P.Re.Val.E. results for 2008-2009 [14] provided an overview of the hospital care heterogeneity in the Lazio region. The results do not constitute a "league table" of performance, but instead reveal numerous instances of high-quality care as well as problem areas that merit further analysis and internal and external auditing as part of an increasingly well-developed program of clinical governance.

P.Re.Val.E. is an outcome research program conceived mainly as a tool for promoting discussion among healthcare managers and professionals in the Lazio region. Given the complexities of accurately comparing provider outcomes, we published the methods used for developing the program in detail, using others' suggestions for the public reporting of comparative health outcome evaluations [47] so that the face validity of the results could be evaluated. We also used various tools to present the results in order to make the results accessible; in particular, we used bar graphs to clearly display adjusted estimates, as well as tables that included the number of admissions, the crude and adjusted estimates, and the statistical significance of the results.

Public disclosure of the 2006-2009 results to clinicians, health care managers, and policy makers was aimed at creating an incentive to improve results. In fact, studies describing the effect of public reporting on consumers' choices, effectiveness, patient safety and patient-centeredness have shown that the public release of performance data mainly stimulates change at hospital level [48]. There have been some negative reactions, but in most cases, the results have stimulated increased review among health care organizations and professionals. Clinicians and program developers have met several times to discuss the methodology as well as negative findings, such as poor performance or poor coding accuracy. Physicians have made suggestions about more accurate selection criteria for some indicators. As a consequence of the extremely low proportion of interventions for hip fracture in the elderly within 48 hours in most facilities, the regional authority decided that hospitals with performance results below a given standard would be penalized economically by a reduction in fees corresponding to specific Diagnosis Related Groups (DRGs) [49].

P.Re.Val.E. is a program in progress, and it will be updated and further developed by the definition and calculation of additional indicators, e.g., those aimed at evaluating health care quality for oncology patients, and by the use of regional drug dispensing registries to more accurately identify patient comorbidities. The impact of health care performance information disclosure to the general public should be evaluated. Evidence suggests that this information has only a limited impact on consumer decision-making [39] since people have limited access to data on health care providers [50]; however, studies suggest that people are interested in comparative information [51,52].

These analyses have explicit limitations, especially with regard to the marked variability in the coding accuracy of current health care information systems. This issue is critical for ensuring accurate risk adjustment, and, correspondingly, reliable comparative quality ratings [53]. In the past, administrative databases have too frequently been used exclusively as tools to claim financial reimbursement for services provided without concern for their roles as epidemiologic sources and as essential instruments for clinical governance. There are some important advantages to routinely collecting administrative data: it is inexpensive to do this, and the data provide information about large populations, do not depend on voluntary participation by individual clinicians and providers, and can be used to predict risk of death with discrimination comparable with that obtained from clinical databases [54]. However, the use of routinely collected administrative data in comparative outcome evaluations has been criticized for the following reasons: there is an absence of clinical information needed to adequately adjust for patients' conditions [55,56]; there is an inability to distinguish between a disease present at admission (a comorbidity, i.e. a true patient risk factor) and one that occurred during the hospital stay (i.e. a complication) [56,57] and some chronic comorbidities, such as hypertension and diabetes, are known to be currently under-reported at admission, mainly in more severely affected patients [58,59]. The first problem could be overcome in the P.Re.Val.E. update for 3 conditions, namely AMI, aortocoronary bypass, and hip fracture, since some clinical information (e.g., systolic blood pressure, ejection fraction, creatinine) have been recently added to the HIS [60]. Moreover, in these 3 conditions, intrinsic illnesses at the time of patients' admissions can be distinguished from complications since "present on admission" (POA) flags have been added to discharge diagnoses [61]. The problem of under-recording is also partially solvable by using prior patient hospitalization records to identify comorbidities independent of patient severity at the current admission [61] as well as emergency department visits to collect additional information about patient risk factors. However, the coding accuracy may differ widely among the facilities [62], and this could lead to biased comparisons. Even though the possibility of gaming of the data in response to the performance evaluation cannot be excluded, previous studies did not find evidence of gaming [63]. Some studies have reported that changes in data accuracy may partially explain quality improvement [64]. However, we did not find relevant changes in recording of co-morbidities in our study population over the years (data not shown). In agreement with previous reports, the prevalence of certain co-morbidities and risk factors was relatively low in our study population, indicating underreporting of co-morbidities and detailed clinical information in the administrative database [65]. However, underreporting was non-differential in the years included in our analysis. Moreover, a previous Italian study [65] assessing clinical performance in cardiac surgery demonstrated that the use of an administrative database provided similar league tables as a more complex specialized database.

Finally, since health care services can only be evaluated by empirical measurements, inevitably there will be errors (systematic and random). This represents a clear limitation of our analysis as well as others of this type. We agree with Shahian et al. that hospital mortality estimates could vary, sometimes widely, based on the different case-selection criteria and statistical methods, leading to divergent inferences about relative hospital performance. Despite these concerns, some findings could be useful to potential users or to facilities [66]. P.Re.Val.E. openly declares the data sources and methods, allowing external review of biases and distortions implicit to the evaluation process. The next P.Re.Val.E. analysis, expected in November 2011, will include improvements in methods and procedures; of course, we cannot say that other biases will not be introduced, simply that they will be different. It is our conviction, however, that P.Re.Val.E. is an important operative tool that should be used to promote clinical and organizational monitoring of health care providers, to support political decision-making processes, and to stimulate a sense of healthy, productive competition aimed at improving healthcare efficacy and equity. We hope that this program will encourage a value often neglected within the Italian NHS: accountability.

## Competing interests

The authors declare that they have no competing interests.

## Authors' contributions

DF conceived the idea, participated in the design, analysis, interpretation of data and have been involved in drafting the manuscript. APB participated in the design, interpretation of data and contributed to the manuscript preparation.

CS conducted the statistical analysis, provided technical support and contributed to the manuscript preparation. MD made substantial contributions to perform the statistical analysis. MS conducted the statistical analysis and provided technical support. AL conducted the statistical analysis and provided technical support. MD participated in the design and interpretation of data. CAP participated in the design, coordination and interpretation of data. All authors read and approved the final manuscript.

## Pre-publication history

The pre-publication history for this paper can be accessed here:

http://www.biomedcentral.com/1472-6963/12/25/prepub

## Supplementary Material

Additional file 1 **Appendix: operative protocol**. 30-Day Mortality Rate after hospital admission for Acute Myocardial Infarction (AMI). Click here for file
