# Supplementary material for: P.Re.Val.E.: outcome research program for the evaluation of health care quality in Lazio, Italy
Source: BMC Health Serv Res. 2012 Jan 27;12:25. doi: 10.1186/1472-6963-12-25 (PMC3276429; doi:10.1186/1472-6963-12-25)
Supplement: Additional file 1 — Appendix: operative protocol. 30-Day Mortality Rate after hospital admission for Acute Myocardial Infarction (AMI). [file 1472-6963-12-25-S1.DOC]

**Appendix: operative protocol**

**30-Day Mortality Rate after hospital admission for Acute Myocardial Infarction (AMI)**

- *Definition*

For analysis by admitting hospital or by area of residence: *proportion of hospitalizations for episodes of AMI in which the patient died within 30 days of hospital admission.*
An episode of AMI includes all hospitalizations and, when present, emergency department (ED) visits that took place within the 4 weeks following the admission date of the first hospitalization for AMI (*index hospitalization*).

-*Numerator*
Number of AMI episodes in which death is reported to have occurred within 30 days of the index hospitalization admission date

- *Denominator*

Number of episodes of AMI

. *Data sources*

Data was collected from the HIS, the EIS, and the MIS.

*Observation periods*

The following periods were defined:

- recruitment period: 1 January 2006-30 November 2009;
- reconstruction of the clinical history: 2 years before the admission date for the index hospitalization;
- follow-up period: 1 month starting from the admission date for the index hospitalization.

**Cohort selection**

*Eligibility criteria*

All acute inpatient admissions to hospitals in the Lazio Region with discharge dates between 1 January 2006 and 30 November 2009 and a main diagnosis of acute myocardial infarction (ICD-9-CM 410.xx) or a main diagnosis compatible with a diagnosis of infarction and a secondary diagnosis of AMI.

The main diagnoses compatible with a diagnosis of infarction, including procedure-related complications, are listed in the following table:

**ICD-9-CM Code**

**Condition**

411 Other acute and subacute forms of ischemic heart disease

413 Angina pectoris

414 Other forms of chronic ischemic heart disease

423.0 Hemopericardium

426 Conduction disorders

427, excluding 427.5 Cardiac dysrhythmias

428 Heart failure

429.5 Rupture of chordae tendineae

429.6 Rupture of papillary muscle

429.71 Acquired cardiac septal defect

429.79 Other sequelae of myocardial infarction, not elsewhere classified (Mural thrombus (atrial) (ventricular) acquired, following myocardial infarction)

429.81 Other disorders of papillary muscle

518.4 Acute edema of lung, unspecified

518.81 Acute respiratory failure

780.01 Coma

780.2 Syncope and collapse

785.51 Cardiogenic shock

799.1 Respiratory arrest

997.02 Iatrogenic cerebrovascular infarction or hemorrhage (postoperative stroke)

998.2 Accidental puncture or laceration during a procedure (accidental perforation by catheter)

*Consecutive exclusion criteria*

- hospitalizations involving nonresidents of the Lazio Region;
- hospitalizations involving patients under 18 years of age or over 100 years of age;
- hospitalizations lasting less than 48 hours and ending in discharge to home or discharge against medical advice;
- hospitalization involving a patient hospitalized with a diagnosis of AMI within the previous 8 weeks;
- transfers from another hospital when there is no record of an index ED access in a hospital other than the one involved in the index hospitalization.

**HIS-EIS database linkage**

A record linkage procedure was used to ascertain ED accesses that occurred before or after the index hospitalization. The former were used to reconstruct the patient’s clinical history, the latter to ensure more complete identification of the diagnoses made during the AMI episode.

**Definition of outcome and ascertainment of vital status**

The adverse outcome was death within 30 days of the admission date for the index hospitalization. Vital status was ascertained from the HIS, the MIS, or the EIS (for accesses subsequent to the index hospitalization).

**Attribution of outcome**

The event was attributed to the hospital where index hospitalization occurred.

**Identification and definitions of chronic comorbidities and other risk / protective factors**
The factors used in the procedure of risk adjustment included: gender (also used for stratified analysis), age, and a series of comorbidities whose presence was assessed during the episode of AMI (within 30 days of the date of index hospitalization), during the index ED access (when present), and in all previous hospitalizations or ED accesses. The index access was defined as the ED access immediately before the index hospitalization (i.e., no more than 1 day before the date of hospital admission).

| **Condition** | **ICD-9-CM code** | |
| --- | --- | --- |
|  | **During the episode of AMI / during index ED access** | **During previous hospitalizations or ED accesses** |
| Cancer | 140.0–208.9 | 140.0–208.9 |
| Diabetes | 250.0-250.9 | 250.0-250.9 |
| Lipid metabolism disturbances | 272 | 272 |
| Obesity | 278.0 | 278.0 |
| Blood disorders | 280-285, 288, 289 | 280-285, 288, 289 |
| Hypertension | 401-405 | 401-405 |
| Previous myocardial infarction | 412 | 410, 412 |
| Other forms of ischemic heart disease |  | 411, 413, 414 |
| Heart failure |  | 428 |
| Ill-defined descriptions or complications of heart disease |  | 429 |
| Rheumatic heart disease | 393-398 | 391, 393-398 |
| Cardiomyopathy | 425 | 425 |
| Acute endocarditis and myocarditis |  | 421, 422 |
| Other heart conditions | 745, V15.1, V42.2, V43.2, V43.3, V45.0 | 745, V15.1, V42.2, V43.2, V43.3, V45.0 |
| Conduction disturbances and arrhythmias |  | 426, 427 |
| Cerebrovascular disease | 433, 437, 438 | 430-432, 433, 434, 436, 437, 438 |
| Vascular disease | 440-448 (escluso 441.1, 441.3, 441.5, 441.6, 444) | 440-448, 557 |
| Chronic obstructive pulmonary disease (COPD) | 491-492, 494, 496 | 491-492, 494, 496 |
| Chronic renal disease | 582-583, 585-588 | 582-583, 585-588 |
| Chronic diseases (liver, pancreas, intestine) | 571-572, 577.1-577.9, 555, 556 | 571-572, 577.1-577.9, 555, 556 |
| Previous coronary artery bypass graft | V45.81 | 36.1, V45.81 |
| Previous coronary angioplasty | V45.82 | 36.0, V45.82 |
| Cerebral revascularization procedures |  | 38.01, 38.02, 38.11, 38.12, 38.31, 38.32 |
| Other cardiac operations |  | 35, 37.0, 37.1, 37.3, 37.4, 37.5, 37.6, 37.9 |
| Other vascular operations |  | 38-39.5, excluding: 38.01, 38.02, 38.5, 38.11, 38.12, 38.31, 38.32, 38.93 |
| Other venous catheterization | 38.93 |  |
